# AGING AND MASS MARKETING FRAUD: EVIDENCE ON REPEAT VICTIMIZATION USING PERPETRATOR DATA

**DOI:** 10.1093/geroni/igad104.0866

**Published:** 2023-12-21

**Authors:** Marguerite DeLiema, Lynn Langton, M Daniel Brannock, Edward Preble

**Affiliations:** University of Minnesota, Twin Cities, Saint Paul, Minnesota, United States; RTI International, Washington, District of Columbia, United States; RTI International, Research Triangle Park, North Carolina, United States; RTI International, Research Triangle Park, North Carolina, United States

## Abstract

Fraud complaint data indicates that adults in their 70s and 80s experience significantly greater fraud losses than middle aged and young adults, however older adults are less likely to self-report victimization overall. One explanation for older adults’ relatively higher financial losses may be that they experience a greater incidence of repeat victimization. In this study, we examine the relationship between age and repeat mass marketing fraud using a novel dataset of nearly one million US mail fraud victims. The data come from four mail fraud enterprises that were investigated by the US Postal Inspection Service and that ran overlapping scams between 2000 and 2018. The perpetrators collected information on victims (name, address, age) and how often they paid money in response to scam solicitations. Datasets were merged, cleaned, and deduplicated. We use a Prentice-Williams-Peterson adaptation of a Cox proportional hazards model to estimate the effect of age on the likelihood of experiencing a subsequent victimization, controlling for the number of prior victimizations. Compared to victims in their 50s, victims aged 70-79 and 80-89 had a 9% greater risk of experiencing another victimization, whereas victims in their 20s had a 24% lower risk of experiencing another victimization. Logistic regression analyses indicate that adults in their 70s and 80s are significantly more likely than those younger than 50 to experience victimization by multiple types of mail scams. This is the first study to use perpetrator data, rather than self-report, to show that older adults face a greater risk of repeat fraud victimization.

